# 531 Comparison of Vashe and Sulfamylon in Reducing Infection in Postoperative Burn Patients: A Cohort Study

**DOI:** 10.1093/jbcr/iraf019.160

**Published:** 2025-04-01

**Authors:** Omar Zein, Hilary Liu, José Arellano, Alain Corcos, Jenny Ziembicki, Yassin Mohamed, Francesco Egro, Garth Elias

**Affiliations:** University of Pittsburgh Medical Center, Mercy Burn Center; University of Pittsburgh Medical Center; University of Pittsburgh Medical Center; University of Pittsburgh Medical Center; University of Pittsburgh Medical Center; University of Pittsburgh; University of Pittsburgh Medical Center; University of Pittsburgh Medical Center

## Abstract

**Introduction:**

Infection is a leading cause of mortality in third-degree burns, contributing to over 75% of burn-related deaths. Vashe and Sulfamylon are commonly used antiseptics in burn care, but comparative data on their efficacy in reducing infection and mortality is limited. This study compares the effectiveness of Vashe and Sulfamylon in managing infections and mortality in postoperative burn patients.

**Methods:**

This retrospective study included adult patients with 3rd-degree burns who underwent surgical excision and grafting from January 2012 to March 2022 at a single ABA-verified burn center. Patients were divided into two groups based on initial antiseptic treatment. Primary outcomes (infection rates, mortality) and secondary outcomes (length of stay, surgical procedures, complications) were compared using chi-square and Mann-Whitney tests.

**Results:**

The study included 138 patients (34.7% female, 65.3% male), predominantly Caucasian (97.8%), with an average age of 51.1 ± 17.7 years and BMI of 28.7 ± 6.5 kg/m². Comorbidities included diabetes (13%), hypertension (30%), and current tobacco use (45%). Most patients (95.6%) sustained thermal burns, while the remaining had electrical, chemical or unknown burn etiology. The average Total Body Surface Area (TBSA) burned was 13.1 ± 15.2%. Of the patients, 89.1% received Sulfamylon and 10.9% Vashe. Mortality was 7.3% in the Sulfamylon group, with no deaths in the Vashe group (p=0.28). The average treatment duration was 12.0 ± 10.6 days for Sulfamylon and 8.0 ± 4.4 days for Vashe (p=0.33). Hospital stays averaged 19.1 ± 14.7 days for Sulfamylon and 21.6 ± 11.1 days for Vashe (p=0.44). Time to surgery averaged 4.4 ± 3.5 days for Sulfamylon and 4.5 ± 2.1 days for Vashe (p=0.57). Patients required 2.1 ± 1.5 operations for Sulfamylon and 1.9 ± 1.9 for Vashe (p=0.41). Infection occurred after 7.7 ± 8.8 days in Sulfamylon and 8.7 ± 1.2 days in Vashe (p=0.31).

**Conclusions:**

No significant differences were found between Sulfamylon and Vashe in infection, mortality or morbidity outcomes. Both treatments showed similar efficacy in infection control, length of stay and surgical procedures.

**Applicability of Research to Practice:**

Both Vashe and Sulfamylon are viable antiseptic options for postoperative burn care, with no significant differences in infection control, mortality, or morbidity outcomes. Clinicians can be flexible in choosing either treatment based on patient needs, cost, or availability without compromising care quality.

**Funding for the Study:**

N/A

